# Parental educational level and childhood wheezing and asthma: A prospective cohort study from the Japan Environment and Children’s Study

**DOI:** 10.1371/journal.pone.0250255

**Published:** 2021-04-16

**Authors:** Yasuaki Saijo, Eiji Yoshioka, Yukihiro Sato, Toshinobu Miyamoto, Hiroshi Azuma, Yusuke Tanahashi, Yoshiya Ito, Sumitaka Kobayashi, Machiko Minatoya, Yu Ait Bamai, Keiko Yamazaki, Sachiko Itoh, Chihiro Miyashita, Atsuko Araki, Reiko Kishi, the Japan Environment and Children’s Study (JECS) Group

**Affiliations:** 1 Division of Public Health and Epidemiology, Department of Social Medicine, Asahikawa Medical University, Asahikawa, Japan; 2 Department of Obstetrics and Gynecology, Asahikawa Medical University, Asahikawa, Japan; 3 Department of Pediatrics, Asahikawa Medical University, Asahikawa, Japan; 4 Faculty of Nursing, Japanese Red Cross Hokkaido College of Nursing, Kitami, Japan; 5 Center for Environmental and Health Sciences, Hokkaido University, Sapporo, Japan; Norwegian Institute of Public Health, NORWAY

## Abstract

**Background:**

The influence of mothers’ and fathers’ educational levels in separate evaluations of asthma has not been fully investigated. This study aims to examine the associations of the mother’s and fathers’ educational levels with childhood wheeze and asthma adjusting for crude and pre-and post-natal modifiable risk factors.

**Methods:**

We conducted a prospective cohort study using data from the Japan Environment and Children’s Study, which recruited pregnant women from 2011 to 2014. The mother’s and father’s educational levels were surveyed by a questionnaire during the pregnancy, and childhood wheezing and doctor-diagnosed asthma were estimated using a 3-year questionnaire. Multilevel logistic regression analysis was performed to evaluate the association between the mother’s and father’s educational levels and childhood wheezing and asthma, adjusted for pre-and post-natal factors.

**Results:**

A total of 69,607 pairs of parents and their single infants were analyzed. We found 17.3% of children had wheezing and 7.7% had asthma. In crude analyses, lower educational level of parents was associated with an increased risk of childhood wheezing and asthma. After full adjustment, a lower educational level of mothers was associated with an increased risk of childhood asthma (junior high school (reference: high school); odds ratio (OR): 1.17, 95% CI, 1.01–1.36), and higher educational level, especially the mother’s, was associated with an increased risk of childhood wheezing (technical junior college, technical/vocational college, or associate degree (ECD3); OR: 1.12, 95% CI, 1.06–1.18, bachelor’s degree, or postgraduate degree; OR: 1.10, 95% CI, 1.03–1.18), and asthma (ECD3; OR: 1.13, 95% CI, 1.04–1.21).

**Conclusions:**

Parents’ lower educational level was a crude risk factor for childhood wheezing and asthma. However, an increased risk of wheezing due to mothers’ higher educational level was found after adjusting for pre-and post-natal factors.

## Introduction

Asthma is a common non-communicable disease affecting people of all ages around the world, and it has a substantial burden of disease like premature death and reduced quality of life [1]. The prevalence of childhood asthma in Japan is at the middle-ranked level in the world, although a very recent survey indicates that asthma prevalence is declining [2]. Wheezing is a frequent symptom for preschool children, which often disappear by the time they reach school age, but some of them remain symptomatic, developing persistent wheezing [3]. Further, adult-onset asthma may be affected by early childhood wheezing with pathogenesis changes [4].

The social determinants of health are one of the principal causes of health inequalities, and parents’ low socioeconomic status negatively influences their child’s physical and mental health [5, 6]. Several studies reported that lower mother’s educational level (no evaluation of father’s educational level) was associated with childhood asthma [7–9], but no association was reported after adjusting for post-natal factors including breastfeeding [10]. Swedish and Dutch studies which defined the parental educational level as the highest education attained within each couple found that lower educational level was associated with significantly higher childhood asthma risk, but the adjustments did not include post-natal factors [11, 12]. A study that measured both mothers’ and fathers’ educational levels reported that only lower paternal educational level had a significant relationship without postnatal factor adjustment [13]. However, one Japanese study reported no significant relationship of both mother’s and father’s educational levels with pre- and postnatal factors adjustment [14].

Thus, parental lower educational attainment broadly links to childhood asthma, but the influence is attenuated by modifiable factors, especially post-natal factors. Furthermore, the influence of mothers’ and fathers’ educational levels on childhood asthma has not been fully investigated. This study aims to examine the crude and pre-and post-natal modifiable risk factors and adjusted associations of mothers’ and fathers’ educational levels with childhood wheezing and asthma by using birth cohort data from the Japan Environment and Children’s Study (JECS) [15, 16].

## Methods

### Participants

The JECS is an ongoing nationwide prospective birth cohort study in Japan that aims to identify environmental factors of children’s health and development [15, 16]. Fifteen regional centers were selected to cover all the Japanese geographical areas (Hokkaido, Miyagi, Fukushima, Chiba, Kanagawa, Koshin, Toyama, Aichi, Kyoto, Osaka, Hyogo, Tottori, Kochi, Fukuoka, and South Kyushu/Okinawa). To maximize representativeness, baseline recruitment was performed in collaboration with local governments and co-operating health care providers. Follow-up of the children from these pregnancies is ongoing until they reach 13 years of age.

**Fig 1:**
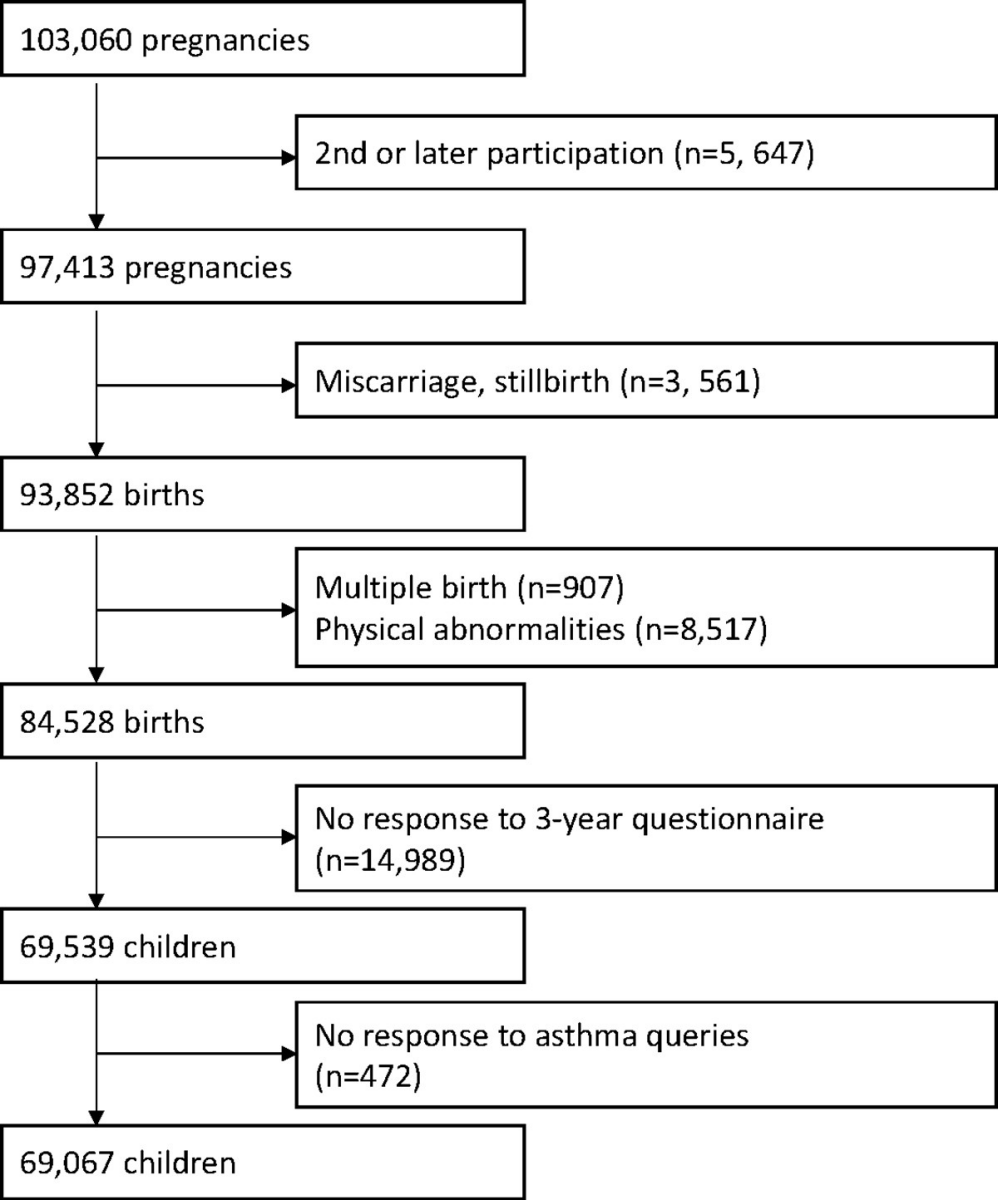
Flowchart of the study.

Pregnant women in the early stages of their pregnancy who resided in the study area were recruited, and a total of 103,060 pregnancies throughout Japan were covered in this study between January 2011 and March 2014. It is difficult to correctly indicate the coverage of the children (the number of live births registered in JECS divided by the number of all live births within the study areas) for the whole study period because we recruited women in early pregnancy and later expanded the study areas. However, in 2013, when recruitment was largely stabilized, the child coverage was approximately 45% [16]. Discounting pregnancies in the same woman, the study involved 97,413 unique pregnancies. After excluding miscarriage, stillbirth, multiple births, positive physical abnormalities (infants’ medical records at one month of age), not responding to the three-year questionnaire (response rate of unique pregnancy without before mentioned exclusion criteria: 82.3%), and not responding to wheezing queries of the International Study of Asthma and Allergies in Childhood (ISAAC) [17–19] in the three-year questionnaire, the number of final analyzed participants was 69,067 children (Fig 1). We used the dataset of jecs-ta-20190930 from the JECS, which is the registry of the JECS is the University Hospital Medical Information Network (UMIN) 000030786 (UMIN Clinical Trials Registry).

### Ethics statement

The JECS protocol was approved by the Institutional Review Board on epidemiological studies of the Ministry of the Environment and by the Ethics Committees of all participating institutions. The JECS was conducted in accordance with the Declaration of Helsinki and other nationally valid regulations. Written informed consent was obtained from all participating mothers and fathers.

### Outcomes

Wheezing (primary outcome) and doctor-diagnosed (secondary outcome: additional analysis) asthma at the 3-year questionnaire were outcomes. In the primary outcome, we used the Japanese translation of the International Study of Asthma and Allergies in Childhood (ISAAC) questionnaire for 6-7-year children [17–19]. Caregivers were asked, “Has your child ever had wheezing or whistling in the chest at any time in the past?” If yes, then they were asked “Has your child had wheezing or whistling in the chest in the last 12 months (yes or no)?" If yes, we defined the children as wheezing positive at 3-years. In the secondary outcome, caregivers were also asked, “Has your child had any diseases which a medical doctor diagnosed since 2-years age?” If they had asthma and we checked whether they had any other diseases or “no diseases,” we defined the children as negative of doctor-diagnosed asthma. If any diseases and diseases were unchecked, the outcome of doctor-diagnosed asthma was considered as a missing value.

### Mother’s and father’s educational levels

Questionnaires were administered to enrolled mothers during the first (T1) and second/third trimester (T2). The T2 questionnaire included questions about the educational attainments of mothers and fathers. They were categorized as ≤ 9 years (junior high school: EDC1), 10–12 years (high school: EDC2), 13–15 years (technical junior college, technical/vocational college, or associate degree: EDC3), or ≥ 16 years (bachelor’s degree or postgraduate degree: EDC4).

### The other independent variables

Based on previous studies [5, 7, 8, 10–14, 20], the following variables were selected as covariates: child sex, gestational age, season of birth, parity, type of delivery, mother and father age, pre-pregnancy body mass index (BMI), household income, marital status, mother and father smoking status during pregnancy, mother and father allergy, breast milk, nursery, lower respiratory infection, mold at home, pet, and passive smoke.

The T1 questionnaire for mothers included questions regarding the mother’s date of birth, marital status, mother and father smoking habit, and past history of mother doctor-diagnosed allergy-related diseases. Marital status was classified as married, unmarried, divorced, or bereavement. Smoking habit was categorized as never smoked, quit smoking before pregnancy, quit smoking during pregnancy, or continued smoking during pregnancy. Mothers responded to the question, “Have you ever been diagnosed by a physician for asthma, allergic rhinitis/hay fever, atopic dermatitis, allergic conjunctivitis, or food allergy (including oral allergy syndrome)?”. We defined anyone with at least one allergic disease as allergy positive. A questionnaire for fathers during pregnancy also included birth day and the past history of doctor-diagnosed allergy-related diseases, and they were categorized in the same manner as mother allergy.

The following information was also collected from medical records transcripts: child’s date of birth, parity, gestational period, height, and pre-pregnancy weight, from which BMI was calculated. Mother and father ages at the time of childbirth were calculated from their birth date and the child’s birth date.

Breastfeeding was categorized as ≤ 1 month, 2 to 5 months, and ≥6 months using a 1-year questionnaire. Using a 3-year questionnaire, nursery admission was defined if the child went to a nursery and was under 2 years of age.

Regarding the child’s environment after birth, mold growth, pet at home, and passive smoke were collected from the 1.5-year questionnaire. Mold growth positive was defined if they answered positive to any of the queries regarding mold growth in the living room, child’s bedroom, or caregiver’s bedroom if the child slept there. Pet at home positive was defined if they had any dogs, cats, small mammals, or birds. Caregivers were asked, “Has anyone smoked around your child?”, and the child’s exposure to passive smoke was categorized as none, sometimes, and often.

The number of lower respiratory infections was defined as adding the positive numbers of 1-year (recall period: from birth), 1.5-year (recall period: from one year old), and 2-year questionnaires (recall period: from 1.5 years old).

### Statistical analysis

The associations between the two outcomes and child’s sex, gestational age, season of birth, parity, type of delivery, mother’s and father’s age, pre-pregnancy BMI, household income, marital status, mother’s and father’s smoking status during pregnancy, mother’s and father’s allergy, breast milk, nursery, lower respiratory infection, mold at home, pet, passive smoke, and mother’s and father’s educational levels were investigated using Fisher’s exact test or chi-square test (supplemental analysis).

To impute missing values for participants with missing data (7.1% of all data except for 15 regional units that had no missing data), the information was replaced using multiple imputations (25 imputed datasets) based on the assumption that data were missing at random. The variables included in the imputation model were as follows: 15 regional centers, wheezing, doctor-diagnosed asthma, child sex, gestational age, season of birth, parity, type of delivery, mother’s and father’s age, pre-BMI, household income, marital status, mother’s and father’s smoking status during pregnancy, mother’s and father’s allergy, breast milk, nursery, lower respiratory infection, mold at home, pet, passive smoke, and mother’s and father’s educational levels. Using the imputed datasets, the crude odds ratios (ORs) of each variable for childhood wheezing and doctor-diagnosed asthma were calculated (supplemental analysis).

Next, we conducted multilevel logistic regression analyses in which individual-level factors were at the first level and 15 regional centers at the second level to estimate ORs for childhood wheezing (primary outcome) and doctor-diagnosed asthma (secondary outcome: additional analysis) with 95% confidence intervals (95% CIs). First, mothers’ and fathers’ educational levels were separately introduced (crude model). In Model 1, mothers’ and fathers’ educational levels was simultaneously introduced. In Model 2, child sex, gestational age, season of birth, parity, type of delivery, mother’s and father’s age, pre-pregnancy BMI, marital status, mother’s and father’s allergy, household income and mother’s and father’s smoking status breast milk, nursery, the number of lower respiratory infections, mold, pet at home, and passive smoke were added. Then, to confirm the combination exposure effects of the mother and father’s educational levels, the combination variable was introduced in crude and full adjusted models (additional analysis).

Two-sided P-values <0.05 were considered statistically significant. All analyses were conducted using Stata statistical software version 15.0 for Windows (StataCorp, College Station, TX, USA).

## Results

Percentages of childhood wheezing and doctor-diagnosed asthma positives at 3-year were 17.4% and 7.6%, respectively (Table 1, S1 Table). Both mother’s and father’s educational levels had statistically significant relationships with wheezing and doctor-diagnosed asthma in the pairwise deletion analyses (S2 and S3 Tables).

**Table 1:** Child sex, mothers’ and fathers’ educational levels, and outcomes. (N = 69,067).

	N	%
Sex		
Boy	35,198	51.0
Girl	33,869	49.0
Mother’s educational level		
EDC1	2,536	3.7
EDC2	20,613	29.8
EDC3	29,354	42.5
EDC4	15,788	22.9
Missing	776	1.1
Father’s educational level		
EDC1	4,263	6.2
EDC2	24,232	35.1
EDC3	15,654	22.7
EDC4	23,783	34.4
Missing	1,135	1.6
3y Wheezing (ISSAC)		
Positive	12,014	17.4
Negative	57,053	82.6
3y Doctor-diagnosed asthma		
Positive	5,249	7.6
Negative	57,310	83.0
Missing	6,508	9.4

Junior high school: EDC1, high school: EDC2, technical junior college, technical/vocational college, or associate degree: EDC3, bachelor’s degree, or postgraduate degree: EDC4.

In crude general logistic regressions analysis of childhood wheezing after multiple imputation among mothers (S4 Table), EDC1 had a significantly higher OR for wheezing (OR 1.12; 95% CI, 1.01–1.30), and EDC3 had a higher OR (OR 1.08; 95% CI, 1.03–1.13), and EDC4 had no significance (reference: EDC2). Among fathers, EDC1 had a significantly higher OR (OR 1.09; 95% CI, 1.01–1.25), and EDC3 (OR 0.94; 95% CI, 0.89–1.99) and EDC4 (OR, 0.87; 95% CI, 0.83–0.92) had lower ORs. For childhood doctor-diagnosed asthma, EDC1 had a significantly higher OR (OR 1.41; 95% CI, 1.22–1.61), and EDC4 (OR 0.83; 95% CI, 0.76–0.90) had lower ORs among mothers. Among fathers, EDC1 had a significantly higher OR (OR 1.15; 95% CI, 1.02–1.28), and EDC3 (OR 0.92; 95% CI, 0.86–1.00) and EDC4 (OR 0.78; 95% CI, 0.72–0.83) had lower ORs. (S5 Table).

### Associations of mothers’ and fathers’ educational levels with childhood wheezing

Table 2 displays the results of a multilevel analysis of the associations of mothers’ and fathers’ educational levels with childhood wheezing. In the crude models, the significance was the same as in the general logistic regression except for the father’s EDC3 (fEDC3). In the final model (Model 2), only mEDC3 and 4 had significantly higher ORs (EDC3; OR 1.12; 95%CI, 1.06–1.18; EDC4: OR, 1.10; 95%CI, 1.03–1.18).

**Table 2:** Crude and adjusted odds ratios for wheezing in multilevel logistic regression analysis (multiple imputation, N = 69,067).

	Crude		Model 1		Model 2	
	OR	95%CI	OR	95%CI	OR	95%CI
Mother’s education						
EDC1	**1.16**	**[1.04, 1.29]**	1.11	[0.996, 1.24]	1.02	[0.91, 1.14]
EDC2	1.00		1.00		1.00	
EDC3	**1.11**	**[1.06, 1.16]**	**1.14**	**[1.09. 1.20]**	**1.12**	**[1.06, 1.18]**
EDC4	1.01	[0.95, 1.07]	**1.07**	**[1.01, 1.14]**	**1.10**	**[1.03, 1.18]**
Father’s education						
EDC1	**1.14**	**[1.05. 1.24]**	**1.15**	**[1.05, 1.25]**	1.04	[0.95, 1.14]
EDC2	1.00		1.00		1.00	
EDC3	0.96	[0.91, 1.02]	**0.94**	**[0.89, 0.996]**	0.96	[0.91, 1.02]
EDC4	**0.91**	**[0.87, 0.96]**	**0.90**	**[0.85, 0.94]**	1.00	[0.94, 1.06]

Junior high school: EDC1, high school: EDC2, technical junior college, technical/vocational college, or associate degree: EDC3, bachelor’s degree, or postgraduate degree: EDC4.

Model 1: Mother’s and father’s education were simultaneously introduced into the model.

Model 2: Model 1 + sex, gestational age at birth, season of birth, type of delivery, parity, mother’s age, father’s age, pre-pregnancy BMI, marital status, mother allergy, and father allergy, household income, mother smoking, father smoking, breast milk, nursery (<2y), mold (1.5y), pet (1.5y), passive smoke (1.5y), and lower respiratory infection were introduced into the model.

### Associations of mothers’ and fathers’ educational level with doctor-diagnosed asthma

Table 3 displays the results of a multilevel analysis of the associations of mothers’ and fathers’ educational level with doctor-diagnosed asthma. In the crude models, the significance was the same as in the usual logistic regression except for f EDC3. In the final model (Model 4), mEDC1 and mEDC3 had a significantly higher OR (EDC1; OR 1.17; 95%CI, 1.01–1.36; EDC3: OR, 1.13; 95%CI, 1.04–1.21).

**Table 3:** Crude and adjusted odds ratios for doctor-diagnosed asthma in multilevel logistic regression analysis (multiple imputation, N = 69,067).

	Crude		Model 1		Model 2	
	OR	95%CI	OR	95%CI	OR	95%CI
Mother’s education						
EDC1	**1.46**	**[1.27, 1.68]**	**1.39**	**[1.21, 1.60]**	**1.17**	**[1.01, 1.36]**
EDC2	1.00		1.00		1.00	
EDC3	1.06	[0.99, 1.14]	**1.10**	**[1.03, 1.18]**	**1.13**	**[1.04, 1.21]**
EDC4	**0.88**	**[0.81, 0.96]**	0.96	[0.88, 1.05]	1.06	[0.96, 1.17]
Father’s education						
EDC1	**1.20**	**[1.07, 1.35]**	**1.16**	**[1.03, 1.30]**	1.01	[0.90, 1.14]
EDC2	1.00		1.00		1.00	
EDC3	0.96	[0.89, 1.03]	0.96	[0.89, 1.03]	0.99	[0.92, 1.08]
EDC4	**0.83**	**[0.77, 0.89]**	**0.85**	**[0.79, 0.91]**	0.97	[0.89, 1.05]

Junior high school: EDC1, high school: EDC2, technical junior college, technical/vocational college, or associate degree: EDC3, bachelor’s degree, or postgraduate degree: EDC4.

Model 1: Mother’s and father’s education were simultaneously introduced into the model.

Model 2: Model 1 + sex, gestational age at birth, season of birth, type of delivery, parity, mother’s age, father’s age, pre-pregnancy BMI, marital status, mother allergy, and father allergy, household income, mother smoking, father smoking, breast milk, nursery (<2y), mold (1.5y), pet (1.5y), passive smoke (1.5y), and lower respiratory infection were introduced into the model.

### Associations of the combination of mother’s and father’s educational level with childhood wheezing

Table 4 displays the results of the multilevel analysis of associations of the combination of mother’s and father’s educational level with childhood wheezing. In crude mode, mEDC1+fEDC1, mEDC2+fEDC1, mEDC3+fEDC1, 2, and 3 had significantly higher ORs, and mEDC2+fEDC4 had a significantly lower OR. In adjusted model, mEDC3+fEDC1 (OR 1.22; 95%CI, 1.04–1.42) and + fEDC 4 (OR, 1.11; 95%CI, 1.02–1.20) had significantly higher ORs.

**Table 4:** Crude and adjusted odds ratios of the combination of mother’s and father’s educational level for wheezing in multilevel logistic regression analysis (multiple imputation, N = 69,067).

			Crude		Adjusted	
		N*	OR	95%CI	OR	95%CI
Mother educational level	Father educational level					
EDC1	EDC1	814	**1.22**	**[1.02, 1.47]**	1.03	[0.84, 1.25]
	EDC2	1,260	1.14	[0.98, 1.32]	1.03	[0.88, 1.21]
	EDC3	301	0.90	[0.65, 1.24]	0.86	[0.61, 1.21]
	EDC4	204	1.05	[0.73, 1.52]	1.04	[0.71, 1.53]
EDC2	EDC1	2,164	**1.14**	**[1.01, 1.29]**	1.02	[0.90, 1.16]
	EDC2	11,026	1.00		1.00	
	EDC3	3,977	0.93	[0.84, 1.02]	0.94	[0.85, 1.04]
	EDC4	3,722	**0.84**	**[0.76, 0.93]**	0.95	[0.85, 1.07]
EDC3	EDC1	1,206	**1.35**	**[1.16, 1.57]**	**1.22**	**[1.04, 1.42]**
	EDC2	9,900	**1.10**	**[1.02, 1.18]**	1.07	[0.99, 1.15]
	EDC3	8,850	**1.08**	**[1.01, 1.17]**	1.08	[0.998, 1.17]
	EDC4	9,705	1.01	[0.94, 1.09]	**1.11**	**[1.02, 1.20]**
EDC4	EDC1	201	0.89	[0.60, 1.34]	0.90	[0.59, 1.36]
	EDC2	2,521	1.07	[0.96, 1.21]	1.13	[0.999, 1.27]
	EDC3	2,757	0.95	[0.85, 1.07]	1.02	[0.90, 1.15]
	EDC4	10,456	0.96	[0.89, 1.03]	1.09	[0.997, 1.18]

* Average number of 25 imputation sets.

Junior high school: EDC1, high school: EDC2, technical junior college, technical/vocational college, or associate degree: EDC3, bachelor’s degree, or postgraduate degree: EDC4.

Adjusted: mother’s and father’s educational levels, gestational age at birth, season of birth, type of delivery, parity, mother’s age, father’s age, pre-pregnancy BMI, marital status, mother allergy, and father allergy, household income, mother smoking, father smoking, breast milk, nursery (<2y), mold (1.5y), pet (1.5y), passive smoke (1.5y), and lower respiratory infection were introduced into the model.

### Associations of the combination of mother’s and father’s educational level with doctor-diagnosed asthma

Table 5 displays the results of the multilevel analysis of associations of the combination of mother’s and father’s educational level with doctor-diagnosed asthma. In crude model, EDC1+fEDC1 and +fEDC2, mEDC2+fEDC1, and mEDC3+ fEDC1 had significantly higher ORs, while mEDC2+fEDC4 and mEDC4+fEDC4 had significantly lower ORs. In adjusted model, only mEDC3+fEDC3 (OR 1.13; 95%CI, 1.01–1.26) had a significantly higher OR.

**Table 5:** Crude and adjusted odds ratios of the combination of mother’s and father’s education for doctor-diagnosed asthma in multilevel logistic regression analysis (multiple imputation, N = 69,067).

			Crude		Adjusted	
		N*	OR	95%CI	OR	95%CI
Mother educational level	Father educational level					
EDC1	EDC1	814	**1.38**	**[1.09, 1.75]**	1.03	[0.80, 1.32]
	EDC2	1,260	**1.46**	**[1.2, 1.77]**	1.22	[0.995, 1.49]
	EDC3	301	1.45	[0.99, 2.12]	1.32	[0.89, 1.97]
	EDC4	204	1.11	[0.66, 1.85]	1.03	[0.61, 1.76]
EDC2	EDC1	2,164	**1.21**	**[1.03, 1.42]**	1.03	[0.87, 1.22]
	EDC2	11,026	1.00		1.00	
	EDC3	3,977	**0.86**	**[0.75, 0.99]**	0.89	[0.77, 1.03]
	EDC4	3,722	**0.80**	**[0.69, 0.93]**	0.95	[0.81, 1.11]
EDC3	EDC1	1,206	**1.32**	**[1.08, 1.62]**	1.22	[0.98, 1.51]
	EDC2	9,900	1.05	[0.95, 1.16]	1.07	[0.96, 1.19]
	EDC3	8,850	1.07	[0.96, 1.18]	**1.13**	**[1.01, 1.26]**
	EDC4	9,705	0.90	[0.81, 1.001]	1.06	[0.94, 1.19]
EDC4	EDC1	201	0.59	[0.30, 1.15]	0.62	[0.31, 1.24]
	EDC2	2,521	0.91	[0.77, 1.07]	1.03	[0.86, 1.22]
	EDC3	2,757	0.87	[0.74, 1.03]	1.02	[0.86, 1.22]
	EDC4	10,456	0.82	[0.74, 0.92]	1.01	[0.89, 1.14]

* Average number of 25 imputation sets.

Junior high school: EDC1, high school: EDC2, technical junior college, technical/vocational college, or associate degree: EDC3, bachelor’s degree, or postgraduate degree: EDC4.

Adjusted: mother’s and father’s educational levels, gestational age at birth, season of birth, type of delivery, parity, mother’s age, father’s age, pre-pregnancy BMI, marital status, mother allergy, and father allergy, household income, mother smoking, father smoking, breast milk, nursery (<2y), mold (1.5y), pet (1.5y), passive smoke (1.5y), and lower respiratory infection were introduced into the model.

## Discussion

This prospective birth cohort study presented associations of lower educational level with increasing childhood wheezing and asthma, and higher educational level with decreasing them in crude analyses, and lower educational level of mothers was associated with increased childhood asthma in the fully adjusted model. However, contrary to expectations, higher educational level, especially the mother’s, was associated with increasing childhood wheezing and asthma after adjusting for pre- and post-natal factors. This is the first study reporting that associations of the combination of mother’ and father’s educational levels on childhood wheezing and asthma in a large-scale prospective birth cohort.

Since lower educational attainment is often linked to limited health literacy and undesirable health behavior [5, 6], the deteriorative effect of lower educational attainment of mother, father, or parents, and the protective effect of higher educational attainment in crude analyses are reported [7–12, 14]. Thus, the results of childhood wheezing and asthma deteriorative effects of lower educational attainment of mothers and fathers, and the protective effect of higher educational attainment in crude analysis in this study are consistent with previous reports. Because early intervention in high-risk groups may reduce asthma development [21], health education for parents risk reduction such as smoking cessation, prevention of exposure to environmental tobacco smoke [22, 23], should be performed from pregnancy.

In a fully adjusted model including pre- and post-natal factors, mother’s EDC3 and 4 were significantly associated with childhood wheezing, and mothers’ EDC1 and EDC3 with childhood asthma. Furthermore, in the fully adjusted model introducing the combination of mother’s and father’s educational levels, mEDC3+fEDC1 and +fEDC4 were significantly associated with childhood wheezing, and mEDC3+fEDC3 with childhood asthma. Two studies evaluated the effect of mothers’ and fathers’ educations simultaneously reported that they had no statistically significant difference after pre- and post-natal factor adjustment [13, 14]. One possible reason for the discrepancy was the difference in the education category between our present study and two previous studies. One previous study categorized education levels as ≤ junior high school and ≥ high school or more (2 categories), the other as primary school, junior high school, high school, and university (4 categories). Thus, two categories of previous Japanese studies may lack detecting differences, which could be clear in 4 categorizations. Because educational level is mandatory through junior high school in Japan, the category in this study started from junior high school. Moreover, the age of childhood in those studies was older than ours (6–7 years and 10 years old). These factors may have caused the discrepancy.

The reasons for higher childhood wheezing and asthma risk among mothers with higher educational attainment after pre- and post-natal factors adjustment could not be elucidated in this study. In the occupation setting, the mental health status of women may not be as good as that of men [24], and for women in Japan, advantaged occupational positions did not link to favorable psychological health or even linked to poor psychological health [25, 26]. However, one study reported that advantaged occupational positions among men had favorable psychological health [26]. An association of maternal psychosocial stress with childhood asthma development was reported, and higher educational attainment has a greater chance to have advanced positions [27]. Thus, the psychosocial stress of mothers with higher educational attainment may be one of the reasons for the higher childhood wheezing and asthma risks.

Another possible factor affecting the significance of mothers’ higher educational level was the difference in reporting rate among mothers’ educational levels. The significant association between mothers’ junior high school education and childhood wheezing disappeared in Model 1, but the association with childhood asthma was retained until Model 2. Mothers with higher education more closely monitor their children’s behaviors and are more health conscientious [28, 29]. Compared to doctor-diagnosed asthma, wheezing involves mild symptoms. This may have caused the report of childhood wheezing among mothers with junior high school education, and the association disappeared in Model 1.

There are several limitations to this study. First, many variables came from self-administered questionnaires. The outcomes may have been underreported, especially mild wheezing, as previously mentioned. Further, doctor-diagnosed asthma was defined based on a self-administered questionnaire to caregivers. Among preschool children, wheezing is very heterogeneous, and the first manifestation of persistent asthma is clinically indistinguishable from transient wheezing. Therefore, doctor-diagnosed asthma was heterogeneous and may have contained transient wheezing [30]. However, we believe doctor-diagnosed asthma was a significant indicator because it reflected severe or recurrent wheezing needing a hospital visit. Second, the generalization of our results to other country contexts may be limited because every nation has an original education system and different rates of entering higher educational institutions. Third, educational attainment could change after childbirth. This may cause educational level misclassification. Fourth, since 16 categories of the combination of the mother’s and father’s educational levels made small number categories including EDC1, the statistical power of those categories was reduced. Fifth, this birth cohort reflected Japanese pregnant women generally [16], but this analysis was limited to those who answered a 3-year questionnaire. Thus, the participants in this study tended to have higher health consciousness. Sixth, the adjustment was made by many known asthma risk factors in our present study, but all possible risk factors were not included (e.g., air pollution). However, major risk factors such as smoking, family history of allergy were included. Furthermore, because the concept of parental educational levels can contain disparities of health literacy, undesirable health behavior, and exposure to contaminants, adjustment of such factors may reduce the comprehensive effect of ‘educational level’ which suggests socio-economic disparity.

## Conclusions

The crude analysis in this prospective birth cohort study demonstrated that mothers’ and fathers’ lower education levels were associated with increased child wheezing and asthma, and higher educational level was associated with decreases in the two outcomes, though mECD3 had a significant crude higher OR for childhood asthma. Pre- and post-natal factor adjustment analysis also indicated that mothers’ lower educational level was associated with increased childhood asthma. Parental educational level to reduce childhood asthma should target mothers and fathers with lower educational attainment. Furthermore, pre- and post-natal factor adjustment analysis, higher educational level, especially mothers’, was associated with increasing childhood wheezing and asthma. Thus, there may be mediating factors that were not included in the full models. Further studies were needed to clarify how the mother’s higher educational level affects childhood asthma, and to elucidate the later year effect of mothers’ and fathers’ educational levels on asthma, including hospitalization and medication.

## Supporting information

S1 Table Characteristics of the participants (full version) (N = 69,067). (DOCX)

S2 Table Relationships between variables and wheezing at 3-year (N = 69,067). (DOCX)

S3 Table Relationships between variables and doctor-diagnosed asthma (N = 62,559). (DOCX)

S4 Table Crude odds ratios for wheezing in logistic regression analysis (multiple imputation, N = 69,067). (DOCX)

S5 Table Crude odds ratios for doctor-diagnosed asthma in logistic regression analysis (multiple imputation, N = 69,067). (DOCX)
