# Supplementary material for: Parental educational level and childhood wheezing and asthma: A prospective cohort study from the Japan Environment and Children’s Study
Source: PLoS One. 2021 Apr 16;16(4):e0250255. doi: 10.1371/journal.pone.0250255 (PMC8051798; doi:10.1371/journal.pone.0250255)
Supplement: S1 Table — (DOCX) [file pone.0250255.s001.docx]

S1 Table. Characteristics of the participants (N=69,067)

|  | N | % |
| --- | --- | --- |
| Sex |  |  |
| Boy | 35,198 | 51.0 |
| Girl | 33,869 | 49.0 |
| Gestational age at birth |  |  |
| <37 weeks | 65,988 | 95.5 |
| 37- | 2,922 | 4.2 |
| Missing | 157 | 0.2 |
| Season of birth |  |  |
| Spring | 16,037 | 23.2 |
| Summer | 18,409 | 26.7 |
| Autumn | 19,045 | 27.6 |
| Winter | 15,576 | 22.6 |
| Type of delivery |  |  |
| Vaginal | 56,217 | 81.4 |
| Cesarean | 12,512 | 18.1 |
| Missing | 338 | 0.5 |
| Parity |  |  |
| 0 | 29,604 | 42.9 |
| 1 | 25,009 | 36.2 |
| >2 | 12,648 | 18.3 |
| Missing | 1,806 | 2.6 |
| 3y Wheezing (ISSAC) |  |  |
| Positive | 12,014 | 17.4 |
| Negative | 57,053 | 82.6 |
| 3y Doctor-diagnosed asthma |  |  |
| Positive | 5,249 | 7.6 |
| Negative | 57,310 | 83.0 |
| Missing | 6,508 | 9.4 |
| Mother’s age (years) |  |  |
| -24 | 5,859 | 8.5 |
| 25-29 | 18,882 | 27.3 |
| 30-34 | 24,937 | 36.1 |
| 35-39 | 16,116 | 23.3 |
| 40- | 3,272 | 4.7 |
| Missing | 1 | 0.0 |
| Father’s age (years) |  |  |
| -24 | 2,020 | 2.9 |
| 25-29 | 8,263 | 12.0 |
| 30-34 | 12,385 | 17.9 |
| 35-39 | 9,615 | 13.9 |
| 40- | 4,909 | 7.1 |
| Missing | 31,875 | 46.2 |
| Pre-pregnancy BMI |  |  |
| -18.4 | 10,718 | 15.5 |
| 18.5-24.9 | 51,308 | 74.3 |
| 25- | 6,995 | 10.1 |
| Missing | 46 | 0.1 |
| Marital status |  |  |
| Married | 65,665 | 95.1 |
| Unmarried | 2,268 | 3.3 |
| Divorced or bereavement | 483 | 0.7 |
| Missing | 651 | 0.9 |
| Mother’s educational level |  |  |
| EDC1 | 2,536 | 3.7 |
| EDC2 | 20,613 | 29.8 |
| EDC3 | 29,354 | 42.5 |
| EDC4 | 15,788 | 22.9 |
| Missing | 776 | 1.1 |
| Father’s educational level |  |  |
| EDC1 | 4,263 | 6.2 |
| EDC2 | 24,232 | 35.1 |
| EDC3 | 15,654 | 22.7 |
| EDC4 | 23,783 | 34.4 |
| Missing | 1,135 | 1.6 |
| Household income (thousand yen/year) |  |  |
| -199 | 3,219 | 4.7 |
| 200-399 | 21,796 | 31.6 |
| 400-599 | 21,478 | 31.1 |
| 600-799 | 10,500 | 15.2 |
| 800-999 | 4,350 | 6.3 |
| 1000- | 2,783 | 4.0 |
| Missing | 4,941 | 7.2 |
| Mother smoking |  |  |
| Non-smoker | 41,389 | 59.9 |
| Ex-smoker who quit before pregnancy | 15,972 | 23.1 |
| Ex-smoker who quit after noticing pregnancy | 8,344 | 12.1 |
| Smoker | 2,529 | 3.7 |
| Missing | 833 | 1.2 |
| Father smoking |  |  |
| Non-smoker | 19,290 | 27.9 |
| Ex-smoker who quit before pregnancy | 16,237 | 23.5 |
| Ex-smoker who quit after noticing pregnancy | 1,645 | 2.4 |
| Smoker | 30,188 | 43.7 |
| Missing | 1,707 | 2.5 |
| Mother allergy |  |  |
| No allergy | 34,208 | 49.5 |
| Allergy | 34,475 | 49.9 |
| Missing | 384 | 0.6 |
| Father allergy |  |  |
| No allergy | 21,091 | 30.5 |
| Allergy | 15,723 | 22.8 |
| Missing | 32,253 | 46.7 |
| Breast milk (month) |  |  |
| <1 | 3,396 | 4.9 |
| 2-5 | 9,253 | 13.4 |
| >6 | 54,687 | 79.2 |
| Missing | 1,731 | 2.5 |
| Nursery (<2y) |  |  |
| No nursery | 37,158 | 53.8 |
| Nursery | 20,408 | 29.6 |
| Missing | 11,501 | 16.7 |
| Lower respiratory infection (times) |  |  |
| 0 | 45,084 | 65.3 |
| 1 | 5,838 | 8.5 |
| 2 | 2,426 | 3.5 |
| 3 | 697 | 1.0 |
| Missing | 15,022 | 21.8 |
| Mold (1.5y) |  |  |
| No mold | 59,586 | 86.3 |
| Mold | 7,093 | 10.3 |
| Missing | 2,388 | 3.5 |
| Pet (1.5y) |  |  |
| No pet | 57,540 | 83.3 |
| Pet | 9,490 | 13.7 |
| Missing | 2,037 | 3.0 |
| Passive smoke (1.5y) |  |  |
| No | 51,297 | 74.3 |
| Sometimes | 12,992 | 18.8 |
| Often | 2,535 | 3.7 |
| Missing | 2,243 | 3.3 |

Junior high school: EDC1, high school: EDC2, technical junior college, technical/vocational college, or associate degree: EDC3, bachelor’s degree, or postgraduate degree: EDC4.
