# Supplementary material for: Parental educational level and childhood wheezing and asthma: A prospective cohort study from the Japan Environment and Children’s Study
Source: PLoS One. 2021 Apr 16;16(4):e0250255. doi: 10.1371/journal.pone.0250255 (PMC8051798; doi:10.1371/journal.pone.0250255)
Supplement: S2 Table — (DOCX) [file pone.0250255.s002.docx]

S2 Table. Relationships between variables and wheezing at 3-year (N=69,067)

|  | No wheezing | | Wheezing | | P |
| --- | --- | --- | --- | --- | --- |
|  | N | % | N | % |  |
| Sex |  |  |  |  | <0.001 |
| Boy | 28,221 | 80.2 | 6,977 | 19.8 |  |
| Girl | 28,832 | 85.1 | 5,037 | 14.9 |  |
| Gestational age at birth |  |  |  |  | <0.001 |
| <37 weeks | 54,670 | 82.9 | 11,318 | 17.2 |  |
| 37- | 2,255 | 77.2 | 667 | 22.8 |  |
| Season of birth |  |  |  |  | <0.001 |
| Spring | 13,335 | 83.2 | 2,702 | 16.9 |  |
| Summer | 15,464 | 84.0 | 2,945 | 16.0 |  |
| Autumn | 15,619 | 82.0 | 3,426 | 18.0 |  |
| Winter | 12,635 | 81.1 | 2,941 | 18.9 |  |
| Type of delivery |  |  |  |  | <0.001 |
| Vaginal | 46,609 | 82.9 | 9,608 | 17.1 |  |
| Cesarean | 10,173 | 81.3 | 2,339 | 18.7 |  |
| Parity |  |  |  |  | <0.001 |
| 0 | 25,083 | 84.7 | 4,521 | 15.3 |  |
| 1 | 20,296 | 81.2 | 4,713 | 18.9 |  |
| >2 | 10,158 | 80.3 | 2,490 | 19.7 |  |
| Mother’s age (years) |  |  |  |  | 0.103* |
| -24 | 4,831 | 82.5 | 1,028 | 17.6 |  |
| 25-29 | 15,589 | 82.6 | 3,293 | 17.4 |  |
| 30-34 | 20,541 | 82.4 | 4,396 | 17.6 |  |
| 35-39 | 13,333 | 82.7 | 2,783 | 17.3 |  |
| 40- | 2,758 | 84.3 | 514 | 15.7 |  |
| Father’s age (years) |  |  |  |  | 0.236* |
| -24 | 1,662 | 82.3 | 358 | 17.7 |  |
| 25-29 | 6,724 | 81.4 | 1,539 | 18.6 |  |
| 30-34 | 10,188 | 82.3 | 2,197 | 17.7 |  |
| 35-39 | 7,911 | 82.3 | 1,704 | 17.7 |  |
| 40- | 4,070 | 82.9 | 839 | 17.1 |  |
| Pre-pregnancy BMI |  |  |  |  | <0.001 |
| -18.4 | 8,953 | 83.5 | 1,765 | 16.5 |  |
| 18.5-24.9 | 42,406 | 82.7 | 8,902 | 17.4 |  |
| 25- | 5,654 | 80.8 | 1,341 | 19.2 |  |
| Marital status |  |  |  |  | 0.029 |
| Married | 54,241 | 82.6 | 11,424 | 17.4 |  |
| Unmarried | 1,899 | 83.7 | 369 | 16.3 |  |
| Divorced or bereavement | 380 | 78.7 | 103 | 21.3 |  |
| Mother’s educational level |  |  |  |  | <0.001 |
| EDC1 | 2,066 | 81.5 | 470 | 18.5 |  |
| EDC2 | 17,126 | 83.1 | 3,487 | 16.9 |  |
| EDC3 | 24,052 | 81.9 | 5,302 | 18.1 |  |
| EDC4 | 13,203 | 83.6 | 2,585 | 16.4 |  |
| Father’s educational level |  |  |  |  | <0.001 |
| EDC1 | 3,433 | 80.5 | 830 | 19.5 |  |
| EDC2 | 19,842 | 81.9 | 4,390 | 18.1 |  |
| EDC3 | 12,950 | 82.7 | 2,704 | 17.3 |  |
| EDC4 | 19,927 | 83.8 | 3,856 | 16.2 |  |
| Household income (thousand yen/year) |  |  |  |  | 0.007* |
| -199 | 2,598 | 80.7 | 621 | 19.3 |  |
| 200-399 | 17,928 | 82.3 | 3,868 | 17.8 |  |
| 400-599 | 17,813 | 82.9 | 3,665 | 17.1 |  |
| 600-799 | 8,719 | 83.0 | 1,781 | 17.0 |  |
| 800-999 | 3,588 | 82.5 | 762 | 17.5 |  |
| 1000- | 2,333 | 83.8 | 450 | 16.2 |  |
| Mother smoking |  |  |  |  | <0.001 |
| Non-smoker | 34,593 | 83.6 | 6,796 | 16.4 |  |
| Ex-smoker who quit before pregnancy | 13,068 | 81.8 | 2,904 | 18.2 |  |
| Ex-smoker who quit after noticing pregnancy | 6,754 | 80.9 | 1,590 | 19.1 |  |
| Smoker | 1,963 | 77.6 | 566 | 22.4 |  |
| Father smoking |  |  |  |  | <0.001 |
| Non-smoker | 16,215 | 84.1 | 3,075 | 15.9 |  |
| Ex-smoker who quit before pregnancy | 13,462 | 82.9 | 2,775 | 17.1 |  |
| Ex-smoker who quit after noticing pregnancy | 1,333 | 81.0 | 312 | 19.0 |  |
| Smoker | 24,647 | 81.7 | 5,541 | 18.4 |  |
| Mother allergy |  |  |  |  | <0.001 |
| No allergy | 29,183 | 85.3 | 5,025 | 14.7 |  |
| Allergy | 27,562 | 80.0 | 6,913 | 20.1 |  |
| Father allergy |  |  |  |  | <0.001 |
| No allergy | 17,528 | 83.1 | 3,563 | 16.9 |  |
| Allergy | 12,720 | 80.9 | 3,003 | 19.1 |  |
| Breast milk (month) |  |  |  |  | 0.043 |
| <1 | 2,797 | 82.4 | 599 | 17.6 |  |
| 2-5 | 7,573 | 81.8 | 1,680 | 18.2 |  |
| >6 | 45,327 | 82.9 | 9,360 | 17.1 |  |
| Nursery (<2y) |  |  |  |  | <0.001 |
| No nursery | 32,203 | 86.7 | 4,955 | 13.3 |  |
| Nursery | 15,887 | 77.9 | 4,521 | 22.2 |  |
| Lower respiratory infection (times) |  |  |  |  | <0.001* |
| 0 | 38,917 | 86.3 | 6,167 | 13.7 |  |
| 1 | 4,044 | 69.3 | 1,794 | 30.7 |  |
| 2 | 1,478 | 60.9 | 948 | 39.1 |  |
| 3 | 365 | 52.4 | 332 | 47.6 |  |
| Mold (1.5y) |  |  |  |  | <0.001 |
| No mold | 49,465 | 83.0 | 10,121 | 17.0 |  |
| Mold | 5,699 | 80.4 | 1,394 | 19.7 |  |
| Pet (1.5y) |  |  |  |  | <0.001 |
| No pet | 47,717 | 82.9 | 9,823 | 17.1 |  |
| Pet | 7,724 | 81.4 | 1,766 | 18.6 |  |
| Passive smoke (1.5y) |  |  |  |  | <0.001 |
| No | 42,805 | 83.5 | 8,492 | 16.6 |  |
| Sometimes | 10,452 | 80.5 | 2,540 | 19.6 |  |
| Often | 2,007 | 79.2 | 528 | 20.8 |  |

*Chi-square test

Junior high school: EDC1, high school: EDC2, technical junior college, technical/vocational college, or associate degree: EDC3, bachelor’s degree, or postgraduate degree: EDC4.
