# Supplementary material for: Parental educational level and childhood wheezing and asthma: A prospective cohort study from the Japan Environment and Children’s Study
Source: PLoS One. 2021 Apr 16;16(4):e0250255. doi: 10.1371/journal.pone.0250255 (PMC8051798; doi:10.1371/journal.pone.0250255)
Supplement: S3 Table — (DOCX) [file pone.0250255.s003.docx]

S3 Table. Relationships between variables and doctor-diagnosed asthma (N=62,559)

|  | No Dr-diagnose asthma | | Dr-diagnosed asthma | | P |
| --- | --- | --- | --- | --- | --- |
|  | N | % | N | % |  |
| Sex |  |  |  |  | <0.001 |
| Boy | 28,827 | 90.0 | 3,187 | 10.0 |  |
| Girl | 28,483 | 93.3 | 2,062 | 6.8 |  |
| Gestational age at birth |  |  |  |  | <0.001 |
| <37 weeks | 54,830 | 91.7 | 4,943 | 8.3 |  |
| 37- | 2,354 | 89.0 | 291 | 11.0 |  |
| Season of birth |  |  |  |  | <0.001 |
| Spring | 13,362 | 92.0 | 1,165 | 8.0 |  |
| Summer | 15,485 | 92.2 | 1,319 | 7.9 |  |
| Autumn | 15,705 | 91.2 | 1,510 | 8.8 |  |
| Winter | 12,758 | 91.0 | 1,255 | 9.0 |  |
| Type of delivery |  |  |  |  | <0.001 |
| Vaginal | 46,783 | 91.8 | 4,162 | 8.2 |  |
| Cesarean | 10,264 | 90.7 | 1,048 | 9.3 |  |
| Parity |  |  |  |  | <0.001 |
| 0 | 24,953 | 92.9 | 1,908 | 7.1 |  |
| 1 | 20,656 | 91 | 2,044 | 9 |  |
| >2 | 10,206 | 89.75 | 1,165 | 10.25 |  |
| Mother’s age (years) |  |  |  |  | <0.001* |
| -24 | 4,669 | 89.4 | 553 | 10.6 |  |
| 25-29 | 15,607 | 91.2 | 1,508 | 8.8 |  |
| 30-34 | 20,776 | 91.9 | 1,837 | 8.1 |  |
| 35-39 | 13,480 | 92.2 | 1,144 | 7.8 |  |
| 40- | 2,778 | 93.1 | 206 | 6.9 |  |
| Father’s age (years) |  |  |  |  | 0.001* |
| -24 | 1,617 | 89.8 | 183 | 10.2 |  |
| 25-29 | 6,760 | 90.8 | 689 | 9.3 |  |
| 30-34 | 10,187 | 91.3 | 970 | 8.7 |  |
| 35-39 | 8,043 | 92.3 | 674 | 7.7 |  |
| 40- | 4,091 | 91.7 | 369 | 8.3 |  |
| Pre-pregnancy BMI |  |  |  |  | <0.001 |
| -18.4 | 8,910 | 91.9 | 788 | 8.1 |  |
| 18.5-24.9 | 42,669 | 91.8 | 3,837 | 8.3 |  |
| 25- | 5,694 | 90.2 | 619 | 9.8 |  |
| Marital status |  |  |  |  | <0.001 |
| Married | 54,559 | 91.7 | 4,948 | 8.3 |  |
| Unmarried | 1,861 | 90.9 | 186 | 9.1 |  |
| Divorced or bereavement | 381 | 86.0 | 62 | 14.0 |  |
| Mother’s educational level |  |  |  |  | <0.001 |
| EDC1 | 1,981 | 88.4 | 261 | 11.6 |  |
| EDC2 | 17,018 | 91.5 | 1,575 | 8.5 |  |
| EDC3 | 24,264 | 91.3 | 2,304 | 8.7 |  |
| EDC4 | 13,453 | 92.9 | 1,027 | 7.1 |  |
| Father’s educational level |  |  |  |  | <0.001 |
| EDC1 | 3,420 | 89.7 | 392 | 10.3 |  |
| EDC2 | 19,885 | 90.9 | 1,993 | 9.1 |  |
| EDC3 | 13,008 | 91.5 | 1,205 | 8.5 |  |
| EDC4 | 20,091 | 92.8 | 1,554 | 7.2 |  |
| Household income (thousand yen/year) |  |  |  |  | <0.001* |
| -199 | 2,591 | 89.3 | 310 | 10.7 |  |
| 200-399 | 17,984 | 91.1 | 1,757 | 8.9 |  |
| 400-599 | 17,871 | 91.9 | 1,572 | 8.1 |  |
| 600-799 | 8,847 | 92.9 | 681 | 7.2 |  |
| 800-999 | 3,650 | 92.3 | 303 | 7.7 |  |
| 1000- | 2,338 | 92.5 | 189 | 7.5 |  |
| Mother smoking |  |  |  |  | <0.001 |
| Non-smoker | 34,835 | 92.4 | 2,887 | 7.7 |  |
| Ex-smoker who quit before pregnancy | 13,070 | 91.2 | 1,263 | 8.8 |  |
| Ex-smoker who quit after noticing pregnancy | 6,801 | 90.3 | 730 | 9.7 |  |
| Smoker | 1,964 | 86.7 | 301 | 13.3 |  |
| Father smoking |  |  |  |  | <0.001 |
| Non-smoker | 16,265 | 92.5 | 1,318 | 7.5 |  |
| Ex-smoker who quit before pregnancy | 13,554 | 92.1 | 1,167 | 7.9 |  |
| Ex-smoker who quit after noticing pregnancy | 1,355 | 91.7 | 123 | 8.3 |  |
| Smoker | 24,771 | 90.8 | 2,503 | 9.2 |  |
| Mother allergy |  |  |  |  | <0.001 |
| No allergy | 28,641 | 93.1 | 2,119 | 6.9 |  |
| Allergy | 28,374 | 90.2 | 3,094 | 9.8 |  |
| Father allergy |  |  |  |  | <0.001 |
| No allergy | 17,477 | 92.1 | 1,504 | 7.9 |  |
| Allergy | 12,930 | 90.6 | 1,349 | 9.5 |  |
| Breast milk (month) |  |  |  |  | <0.001 |
| <1 | 2,784 | 90.0 | 308 | 10.0 |  |
| 2-5 | 7,589 | 90.4 | 810 | 9.6 |  |
| >6 | 45,619 | 92.0 | 3,971 | 8.0 |  |
| Nursery (<2y) |  |  |  |  | <0.001 |
| No nursery | 31,308 | 93.7 | 2,122 | 6.4 |  |
| Nursery | 16,662 | 89.6 | 1,945 | 10.5 |  |
| Lower respiratory infection (times) |  |  |  |  | <0.001* |
| 0 | 39,315 | 94.5 | 2304 | 5.5 |  |
| 1 | 4,614 | 84.8 | 829 | 15.2 |  |
| 2 | 1,787 | 78.4 | 493 | 21.6 |  |
| 3 | 454 | 67.8 | 216 | 32.2 |  |
| Mold (1.5y) |  |  |  |  | 0.245 |
| No mold | 49,463 | 91.8 | 4,446 | 8.3 |  |
| Mold | 5,996 | 91.3 | 569 | 8.7 |  |
| Pet (1.5y) |  |  |  |  | 0.001 |
| No pet | 47,947 | 91.9 | 4,256 | 8.2 |  |
| Pet | 7,782 | 90.7 | 794 | 9.3 |  |
| Passive smoke (1.5y) |  |  |  |  | <0.001 |
| No | 43,014 | 92.2 | 3,647 | 7.8 |  |
| Sometimes | 10,529 | 90.2 | 1,142 | 9.8 |  |
| Often | 2,023 | 89.2 | 246 | 10.8 |  |

*Chi-square test

Junior high school: EDC1, high school: EDC2, technical junior college, technical/vocational college, or associate degree: EDC3, bachelor’s degree, or postgraduate degree: EDC4.
