# Supplementary material for: Parental educational level and childhood wheezing and asthma: A prospective cohort study from the Japan Environment and Children’s Study
Source: PLoS One. 2021 Apr 16;16(4):e0250255. doi: 10.1371/journal.pone.0250255 (PMC8051798; doi:10.1371/journal.pone.0250255)
Supplement: S4 Table — (DOCX) [file pone.0250255.s004.docx]

S4 Table. Crude odds ratios for wheezing in logistic regression analysis (multiple imputation, N=69,067)

|  | OR | 95%CI |
| --- | --- | --- |
| Sex |  |  |
| Boy | 1.00 |  |
| Girl | 0.71 | [0.68, 0.74] |
| Gestational age at birth |  |  |
| <37 weeks | 1.43 | [1.31, 1.56] |
| 37- | 1.00 |  |
| Season of birth |  |  |
| Spring | 1.00 |  |
| Summer | 0.94 | [0.89, 1.00] |
| Autumn | 1.08 | [1.02, 1.14] |
| Winter | 1.15 | [1.08, 1.22] |
| Type of delivery |  |  |
| Vaginal | 1.00 |  |
| Cesarean | 1.12 | [1.06, 1.17] |
| Parity |  |  |
| 0 | 1.00 |  |
| 1 | 1.28 | [1.23, 1.34] |
| >2 | 1.36 | [1.29, 1.44] |
| Mother age |  |  |
| -24 | 1.00 |  |
| 25-29 | 0.99 | [0.92, 1.07] |
| 30-34 | 1.01 | [0.93, 1.08] |
| 35-39 | 0.98 | [0.91, 1.06] |
| 40- | 0.88 | [0.78, 0.98] |
| Father age |  |  |
| -24 | 1.00 |  |
| 25-29 | 1.01 | [0.91, 1.12] |
| 30-34 | 0.97 | [0.88, 1.07] |
| 35-39 | 0.96 | [0.87, 1.06] |
| 40- | 0.92 | [0.82, 1.03] |
| Pre-pregnancy BMI |  |  |
| -18.4 | 1.00 |  |
| 18.5-24.9 | 1.07 | [1.01, 1.13] |
| 25- | 1.20 | [1.11, 1.30] |
| Marital status |  |  |
| Married | 1.00 |  |
| Unmarried | 0.93 | [0.83, 1.04] |
| Divorced or bereavement | 1.28 | [1.03, 1.60] |
| Mother's educational level |  |  |
| EDC1 | **1.12** | **[1.01, 1.25]** |
| EDC2 | 1.00 |  |
| EDC3 | **1.08** | **[1.03, 1.13]** |
| EDC4 | 0.96 | [0.91, 1.01] |
| Father's educational level |  |  |
| EDC1 | **1.09** | **[1.01, 1.19]** |
| EDC2 | 1.00 |  |
| EDC3 | **0.94** | **[0.89, 0.99]** |
| EDC4 | **0.87** | **[0.83, 0.92]** |
| Household income (thousand yen/year) |  |  |
| -199 | 1.00 |  |
| 200-399 | 0.90 | [0.82, 0.99] |
| 400-599 | 0.86 | [0.78, 0.94] |
| 600-799 | 0.85 | [0.77, 0.94] |
| 800-999 | 0.89 | [0.79, 1.00] |
| 1000- | 0.81 | [0.71, 0.92] |
| Mother smoking |  |  |
| Non-smoker | 1.00 |  |
| Ex-smoker who quit before pregnancy | 1.13 | [1.08, 1.19] |
| Ex-smoker who quit after noticing pregnancy | 1.20 | [1.13, 1.27] |
| Smoker | 1.47 | [1.33, 1.61] |
| Father smoking |  |  |
| Non-smoker | 1.00 |  |
| Ex-smoker who quit before pregnancy | 1.09 | [1.03, 1.15] |
| Ex-smoker who quit after noticing pregnancy | 1.23 | [1.08, 1.40] |
| Smoker | 1.19 | [1.13, 1.25] |
| Mother allergy |  |  |
| No allergy | 1.00 |  |
| Allergy | 1.46 | [1.40, 1.52] |
| Father allergy |  |  |
| No allergy | 1.00 |  |
| Allergy | 1.16 | [1.10, 1.23] |
| Breast milk (month) |  |  |
| <1 | 1.00 |  |
| 2-5 | 1.03 | [0.93, 1.14] |
| >6 | 0.96 | [0.88, 1.05] |
| Nursery (<2y) |  |  |
| No nursery | 1.00 |  |
| Nursery | 1.86 | [1.78, 1.94] |
| Lower respiratory infection (per 1-time increase) | 2.06 | [2.00, 2.12] |
| Mold (1.5y) |  |  |
| No mold | 1.00 |  |
| Mold | 1.19 | [1.12, 1.27] |
| Pet (1.5y) |  |  |
| No pet | 1.00 |  |
| Pet | 1.11 | [1.05, 1.18] |
| Passive smoke (1.5y) |  |  |
| No | 1.00 |  |
| Sometimes | 1.23 | [1.17, 1.29] |
| Often | 1.32 | [1.20, 1.46] |

Junior high school: EDC1, high school: EDC2, technical junior college, technical/vocational college, or associate degree: EDC3, bachelor’s degree, or postgraduate degree: EDC4.
