# Supplementary material for: Parental educational level and childhood wheezing and asthma: A prospective cohort study from the Japan Environment and Children’s Study
Source: PLoS One. 2021 Apr 16;16(4):e0250255. doi: 10.1371/journal.pone.0250255 (PMC8051798; doi:10.1371/journal.pone.0250255)
Supplement: S5 Table — (DOCX) [file pone.0250255.s005.docx]

S5 Table. Crude odds ratios for doctor-diagnosed asthma in logistic regression analysis (multiple imputation, N=69,067)

|  | OR | 95%CI |
| --- | --- | --- |
| Sex |  |  |
| Boy | 1.00 |  |
| Girl | 0.66 | [0.62, 0.70] |
| Gestational age at birth |  |  |
| <37 weeks | 1.35 | [1.19, 1.53] |
| 37- | 1.00 |  |
| Season of birth |  |  |
| Spring | 1.00 |  |
| Summer | 0.98 | [0.90, 1.06] |
| Autumn | 1.10 | [1.02, 1.19] |
| Winter | 1.12 | [1.03, 1.22] |
| Type of delivery |  |  |
| Vaginal | 1.00 |  |
| Cesarean | 1.14 | [1.06, 1.22] |
| Parity |  |  |
| 0 | 1.00 |  |
| 1 | 1.28 | [1.20, 1.36] |
| >2 | 1.47 | [1.37, 1.59] |
| Mother age |  |  |
| -24 | 1.00 |  |
| 25-29 | 0.83 | [0.75, 0.92] |
| 30-34 | 0.76 | [0.68, 0.84] |
| 35-39 | 0.74 | [0.67, 0.82] |
| 40- | 0.64 | [0.54, 0.76] |
| Father age |  |  |
| -24 | 1.00 |  |
| 25-29 | 0.91 | [0.79, 1.04] |
| 30-34 | 0.83 | [0.73, 0.95] |
| 35-39 | 0.75 | [0.66, 0.86] |
| 40- | 0.76 | [0.65, 0.89] |
| Pre-pregnancy BMI |  |  |
| -18.4 | 1.00 |  |
| 18.5-24.9 | 1.01 | [0.93, 1.10] |
| 25- | 1.22 | [1.10, 1.37] |
| Marital status |  |  |
| Married | 1.00 |  |
| Unmarried | 1.11 | [0.95, 1.29] |
| Divorced or bereavement | 1.76 | [1.35, 2.30] |
| Mother educational level |  |  |
| EDC1 | **1.41** | **[1.22, 1.61]** |
| EDC2 | 1.00 |  |
| EDC3 | 1.03 | [0.96, 1.10] |
| EDC4 | **0.83** | **[0.76, 0.90]** |
| Father educational level |  |  |
| EDC1 | **1.15** | **[1.02, 1.28]** |
| EDC2 | 1.00 |  |
| EDC3 | **0.92** | **[0.86, 1.00]** |
| EDC4 | **0.78** | **[0.72, 0.83]** |
| Household income (thousand yen/year) |  |  |
| -199 | 1.00 |  |
| 200-399 | 0.82 | [0.72, 0.94] |
| 400-599 | 0.73 | [0.64, 0.83] |
| 600-799 | 0.64 | [0.56, 0.74] |
| 800-999 | 0.70 | [0.59, 0.83] |
| 1000- | 0.67 | [0.56, 0.82] |
| Mother smoking |  |  |
| Non-smoker | 1.00 |  |
| Ex-smoker who quit before pregnancy | 1.16 | [1.09, 1.25] |
| Ex-smoker who quit after noticing pregnancy | 1.29 | [1.18, 1.40] |
| Smoker | 1.82 | [1.61, 2.07] |
| Father smoking |  |  |
| Non-smoker | 1.00 |  |
| Ex-smoker who quit before pregnancy | 1.06 | [0.98, 1.15] |
| Ex-smoker who quit after noticing pregnancy | 1.12 | [0.92, 1.35] |
| Smoker | 1.25 | [1.16, 1.33] |
| Mother allergy |  |  |
| No allergy | 1.00 |  |
| Allergy | 1.47 | [1.39, 1.56] |
| Father allergy |  |  |
| No allergy | 1.00 |  |
| Allergy | 1.21 | [1.11, 1.30] |
| Breast milk (month) |  |  |
| <1 | 1.00 |  |
| 2-5 | 0.96 | [0.84, 1.10] |
| >6 | 0.78 | [0.69, 0.88] |
| Nursery (<2y) |  |  |
| No nursery | 1.00 |  |
| Nursery | 1.71 | [1.60, 1.83] |
| Lower respiratory infection (per 1-time increase) | 2.16 | [2.08, 2.24] |
| Mold (1.5y) |  |  |
| No mold | 1.00 |  |
| Mold | 1.06 | [0.97, 1.16] |
| Pet (1.5y) |  |  |
| No pet | 1.00 |  |
| Pet | 1.14 | [1.05, 1.24] |
| Passive smoke (1.5y) |  |  |
| No | 1.00 |  |
| Sometimes | 1.27 | [1.18, 1.36] |
| Often | 1.42 | [1.24, 1.63] |

Junior high school: EDC1, high school: EDC2, technical junior college, technical/vocational college, or associate degree: EDC3, bachelor’s degree, or postgraduate degree: EDC4.
